# 4D Flow MRI in the portal venous system: imaging and analysis methods, and clinical applications

**DOI:** 10.1007/s11547-022-01553-x

**Published:** 2022-09-19

**Authors:** Ryota Hyodo, Yasuo Takehara, Shinji Naganawa

**Affiliations:** 1 Department of Radiology, Nagoya University Graduate School of Medicine, 65 Tsurumai-cho, Showa-ku, Nagoya, 466-8550 Japan; 2 Department of Fundamental Development for Advanced Low Invasive Diagnostic Imaging, Nagoya University Graduate School of Medicine, Nagoya, Japan

**Keywords:** 4D Flow MRI, Portal venous system, Hemodynamics, Cirrhosis, Portal hypertension, Liver transplantation

## Abstract

Thus far, ultrasound, CT, and 2D cine phase-contrast MRI has been adopted to evaluate blood flow and vascular morphology in the portal venous system; however, all these techniques have some shortcomings, such as limited field of view and difficulty in accurately evaluating blood flow. A new imaging technique, namely 3D cine phase-contrast (4D Flow) MRI, can acquire blood flow data of the entire abdomen at once and in a time-resolved manner, allowing visual, quantitative, and comprehensive assessment of blood flow in the portal venous system. In addition, a retrospective blood flow analysis, i.e., "retrospective flowmetry," is possible. Although the development of 4D Flow MRI for the portal system has been delayed compared to that for the arterial system owing to the lower flow velocity of the portal venous system and the presence of respiratory artifacts, several useful reports have recently been published as the technology has advanced. In the first part of this narrative review article, technical considerations of image acquisition and analysis methods of 4D Flow MRI for the portal venous system and the validations of their results are described. In the second part, the current clinical application of 4D Flow MRI for the portal venous system is reviewed.

**Supplementary Information:**

The online version contains supplementary material available at 10.1007/s11547-022-01553-x.

## Introduction

3D cine phase-contrast (4D Flow) MRI is a new technique that allows comprehensive and retrospective assessments of whole spatio-temporal velocity vectors within the field of view (FOV) [1, 2]. Many studies and clinical applications have already been reported for the heart and aortic regions [3, 4]; however, studies on the portal venous (PV) area are hampered by its complex dual blood supply, respiratory movements, and lower flow velocity. Nevertheless, recent technological innovations [5–26] have facilitated sporadic, if not comprehensive, reports in this sector. This narrative review article discusses the technical and clinical aspects of 4D Flow MRI in the portal system.

## Conventional imaging tools for portal flowmetry

Doppler ultrasonography (US) remains the first choice for evaluating blood flow in the PV system under many conditions [27–30]. It allows portal flow assessments, including velocity and flow direction. Although US is a low-cost and convenient technique, it has several drawbacks [5–9, 20–22, 28–32], such as relatively poor reproducibility, narrow FOV, and limited ability to depict complex structures. Moreover, its image quality is degraded by gases in the gastrointestinal tract and by subcutaneous and organ fat in obese individuals.

Conventional MR flowmetry includes 2D cine phase-contrast (PC) MRI [9, 32–36]. Although 2D cine PC is superior to 4D Flow MRI in terms of temporal and in-plane resolution, a flow measurement plane must be prescribed during MR imaging [8, 21, 36], which is cumbersome and requires operators to have a detailed anatomical understanding. Unlike conventional 2D cine PC or US, 4D Flow MRI can retrieve whole spatio-temporal velocity data en bloc; therefore, retrospective and comprehensive analyses are possible even after patients leave the MR suite.

Dynamic contrast-enhanced (DCE) CT can robustly assess the vascular geometry because it is acquired during a single breath hold, provides high-resolution images, and allows comprehensive morphological analyses [37]. However, its capabilities in flow analysis are limited. Contrast administration is almost compulsory; therefore, patients with allergies or renal dysfunction are not indicated. In addition, CT is not free from radiation exposure [7, 17].

Angiography is relatively invasive, requires contrast media, and involves ionizing radiation; it is no longer used for testing alone except for concurrent interventional procedures [37].

## Technical considerations of 4D Flow MRI in the portal system

### Data acquisition

**Fig. 1:**
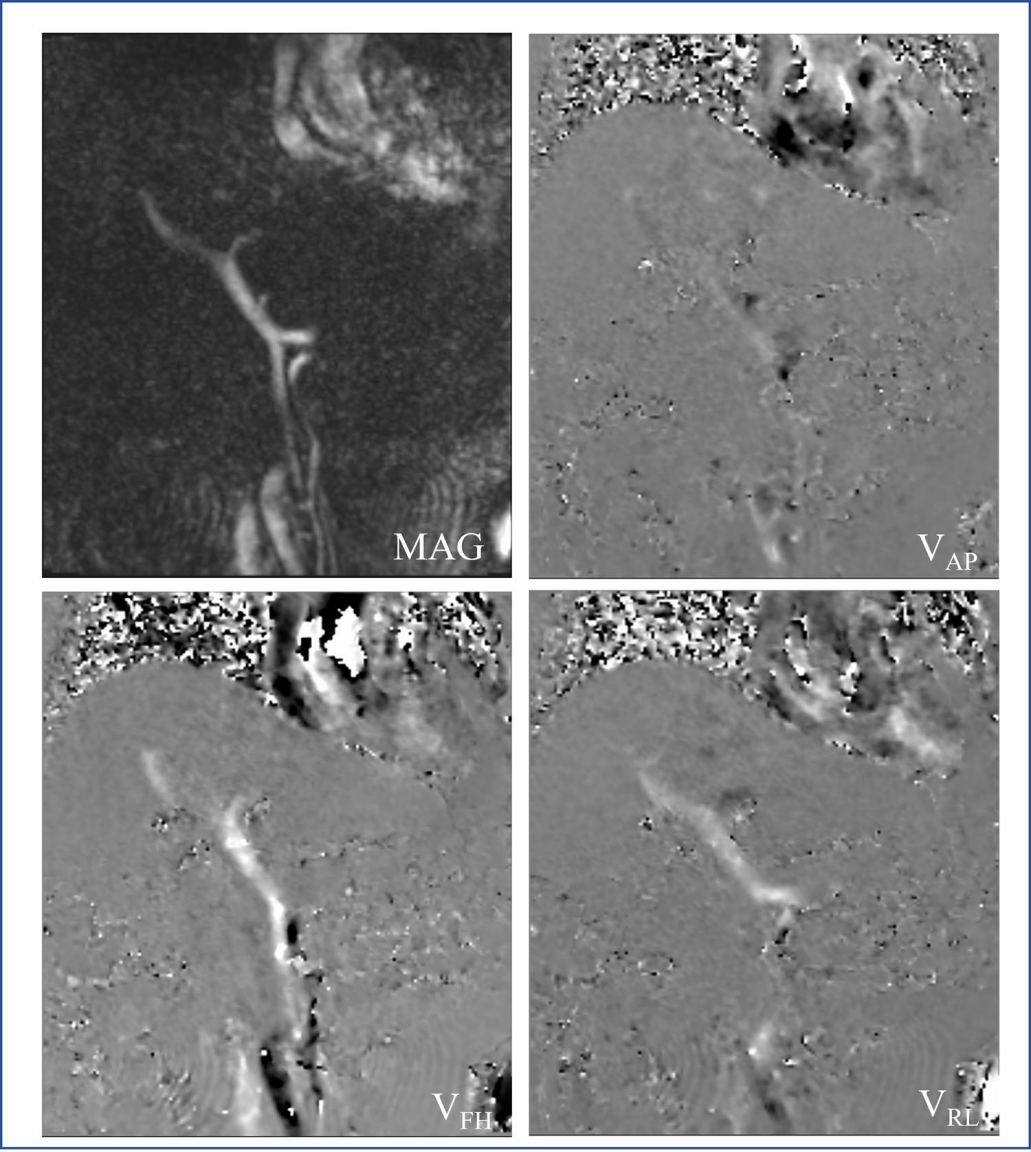
Coronal magnitude image and phase images in three directions. The vector distribution in 3D space is calculated and displayed from the phase images in three directions: anterior-posterior, foot-head, and right-left. *MAG*, magnitude image; *V*_*AP*_, phase image in anterior-posterior direction; *V*_*FH*_, phase image in foot-head direction; *V*_*RL*_, phase image in right-left direction

In 4D Flow MRI, bipolar velocity-encoded gradients are added in three directions (*x*, *y*, and *z*) to obtain three-dimensional velocity vector data (Fig. 1). Protons moving along each axis accumulate a phase shift relative to the stationary protons. The phase shift during a time period corresponds to the velocity, which is displayed as the signal intensity on the phase images.

For row data or k-space trajectories, there are two types of sampling, i.e., Cartesian sampling [5, 7, 10, 23–26] and non-Cartesian sampling. Traditional Cartesian sampling requires around 15–30 min for imaging in the body, whereas further use of cutting-edge techniques allows single-breath-hold acquisition in around 22 s [12, 14]. Non-Cartesian sampling aims to image a larger area in a shorter time and with a higher spatial resolution, and it has been reported for radial [6, 8, 11, 13, 20] and spiral [12, 14] undersampling accelerated acquisition methods. To further reduce the imaging time, methods such as parallel imaging, compressed sensing, and k-t acceleration are often used together [12, 21–25, 38].

Data acquisitions of 4D Flow MRI are synchronized to the cardiac cycle using an electrocardiogram (ECG) or peripheral pulse wave. The two main methods of synchronization are prospective gating [5, 7, 9, 26], in which the imaging begins when the R wave is detected, and retrospective gating [6, 8, 13, 23–25], in which the data are collected continuously, independently of the R wave, and reordered after data completion. Retrospective gating is useful in cases where the R–R intervals are unstable, and the imaging time can be shortened by reducing the amount of data rejected. The Society for Cardiovascular Magnetic Resonance recommends retrospective gating [38]. In the PV area, the number of time frames during one cardiac cycle is often around 10–15 frames/R–R interval [6, 8, 11, 16, 23–25, 39], which is lower than that in the arterial system, because little pulsation occurs in the portal system. Given the lack of pulsation, it may also be reasonable to collect and evaluate time-averaged images without time-resolving if only the portal system is to be evaluated. Landgraf et al. reported that time-averaged reconstruction allows imaging within 3–4 min and yields significantly better results in terms of portal flow measurement accuracy, average streamline length, and visualization quality compared to time-resolved reconstruction [13]. However, time-averaged reconstruction cannot simultaneously evaluate arteries and veins in the vicinity of the PV area and may experience difficulty in reflecting fluctuations due to respiration (Fig. 10).

Adapting a respiratory synchronization program may benefit abdominal imaging. 4D Flow MRI uses navigator echo techniques [5–9, 11, 13, 19–21, 23–26] that place the navigator at the liver–lung interface or the splenic–lung interface, often with a 4–7 mm end-expiratory acceptance window for data acquisition. Spiral trajectories can be imaged with a single breath hold and do not require respiratory gating [12, 14]; however, only blood flow under breath hold can be analyzed, and hemodynamic differences from free breathing must be considered [32, 40–42]. A recently reported self-navigation method collects data from all respiratory phases, allowing imaging in a shorter time regardless of the patient's respiratory status [43].

Maintaining a high signal-to-noise ratio (SNR) is also essential for accurate velocity measurements in 4D Flow MRI. The facilitated method uses the appropriate velocity encoding (VENC) settings and gadolinium contrast agents, which increases the image quality and analytical parameter accuracy [3, 44]. The use of non-specific extracellular contrast agents has been reported in many cases [6, 8, 11–13, 19, 21, 23–25, 39], while the use of hepatocellular-specific contrast agents has also been reported [12]. Post-contrast MR angiography (MRA), e.g., 3D fast spoiled gradient echo-based sequence, as a morphological imaging technique, provides defined vessel wall boundaries, resulting in more accurate segmentation and blood flowmetry [3, 23–25]. For non-contrast morphological images that are to be imaged separately, the use of a flow-sensitive technique, i.e., 3D balanced steady-state free precession imaging, has been reported [24]. Although 3D PC angiography using velocity data or magnitude data is inferior in terms of image quality, it does not require separate morphological imaging, which reduces the examination time and prevents misalignment with the blood flow signal [6–9, 11, 13, 14, 20].

**Fig. 2:**
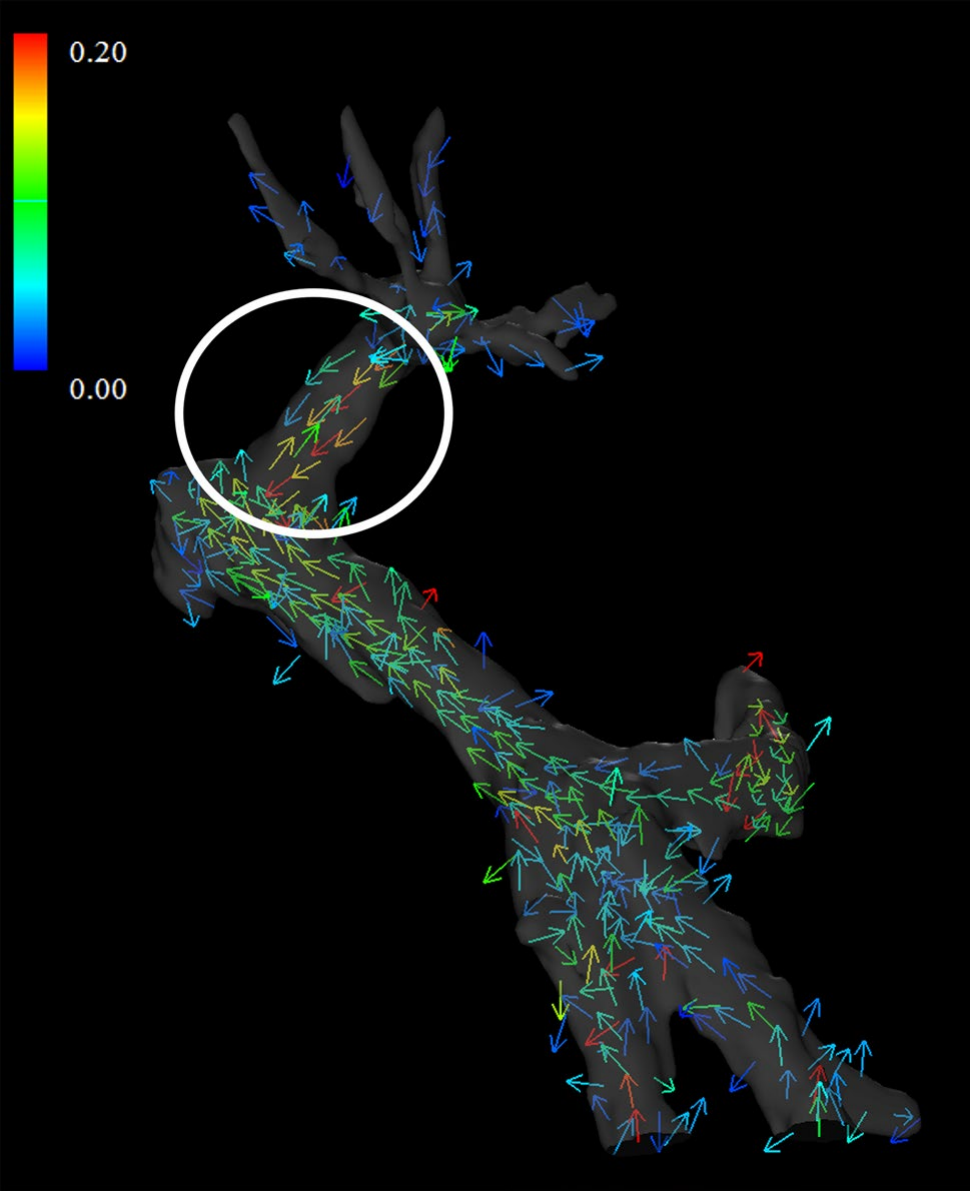
Velocity aliasing. A woman in her 70s after embolization of the right portal vein. The main portal vein flows in an antegrade fashion; however, the left portal vein is retrograde (circle). This is a finding of aliasing because the velocity encoding is too low for the increased flow velocity of the left portal vein after embolization

Optimization of the VENC is essential for accurate blood flow assessment [2]. To enhance the SNR, the VENC should be set to the smallest value that does not exceed the maximum flow velocity of the target vessel. For regions where the flow velocity is higher than the VENC, velocity aliasing occurs, and accurate velocity evaluation becomes difficult (Fig. 2) [2]. Previous reports on normal PV evaluation have shown favorable analytical results with VENC settings of 30–60 cm/s [5, 6, 12, 13, 21]. However, higher VENC settings are necessary in the case of a stenosis because of the increased flow velocity [17, 19, 20, 25]. Higher VENC settings are also required if the arterial and transjugular intrahepatic portosystemic shunt (TIPS) pathways are to be evaluated, in which case the accuracy of the low-flow portal and venous flow parameters is reduced. Another method for optimizing the VENC is to estimate the maximum flow velocity by performing a brief 2D cine PC MRI before 4D Flow MRI. We make it a rule to determine the VENC for 4D Flow MRI by adding 20% to the measured maximum velocity as a safety margin [2, 23, 24, 45].

Dietary intake increases blood flow from the intestinal tract via the superior mesenteric vein (SMV). Therefore, it is conducive to portal blood flow measurements [11, 16, 46]. Previous reports have shown that the time between dietary intake and examination and the dietary content (e.g., fat-rich meals tend to increase blood flow more than carbohydrate-rich meals) affect the portal venous flow [46]. Therefore, 4D Flow MRI is often performed with fasting to exclude the influence of meals. However, the duration of fasting in previous reports has not been consistent, ranging from 2 to 6 h [8, 11, 12, 14, 16, 23–26]. In addition to dietary effects, some herbal medicines increase intestinal blood flow [47].

### Data analysis and visualization

Segmentation of the blood vessels for analysis is performed from the acquired morphological image data. In many previous studies, 3D PC MRA was conducted using phase images and subsequently adopted as morphological images for segmentation [5–9, 11, 13, 14, 16, 18, 26]. However, 3D PC MRA depends on the velocity vector distribution in the vessel in 4D Flow MRI, which often results in unsatisfactory image quality compared to imaging the vessel morphology in a separate sequence. Therefore, we perform MRA separately as described above. Unlike arteries, segmentation of the PV area and veins has the following disadvantages: evaluation based on the signal value threshold is complex and the operator's subjectivity affects the detection of the vessels and contours to some extent. Thus, the size and morphology of the vessels may be inconsistent among researchers. The errors that occur here may affect the flow rate. Therefore, it is important to perform evaluations with the same criteria and pay attention to the variations among researchers. Segmentation of morphological images using artificial intelligence (AI) technology has been reported for the aorta [48], and future adaptations are expected for the PV area.

**Fig. 3:**
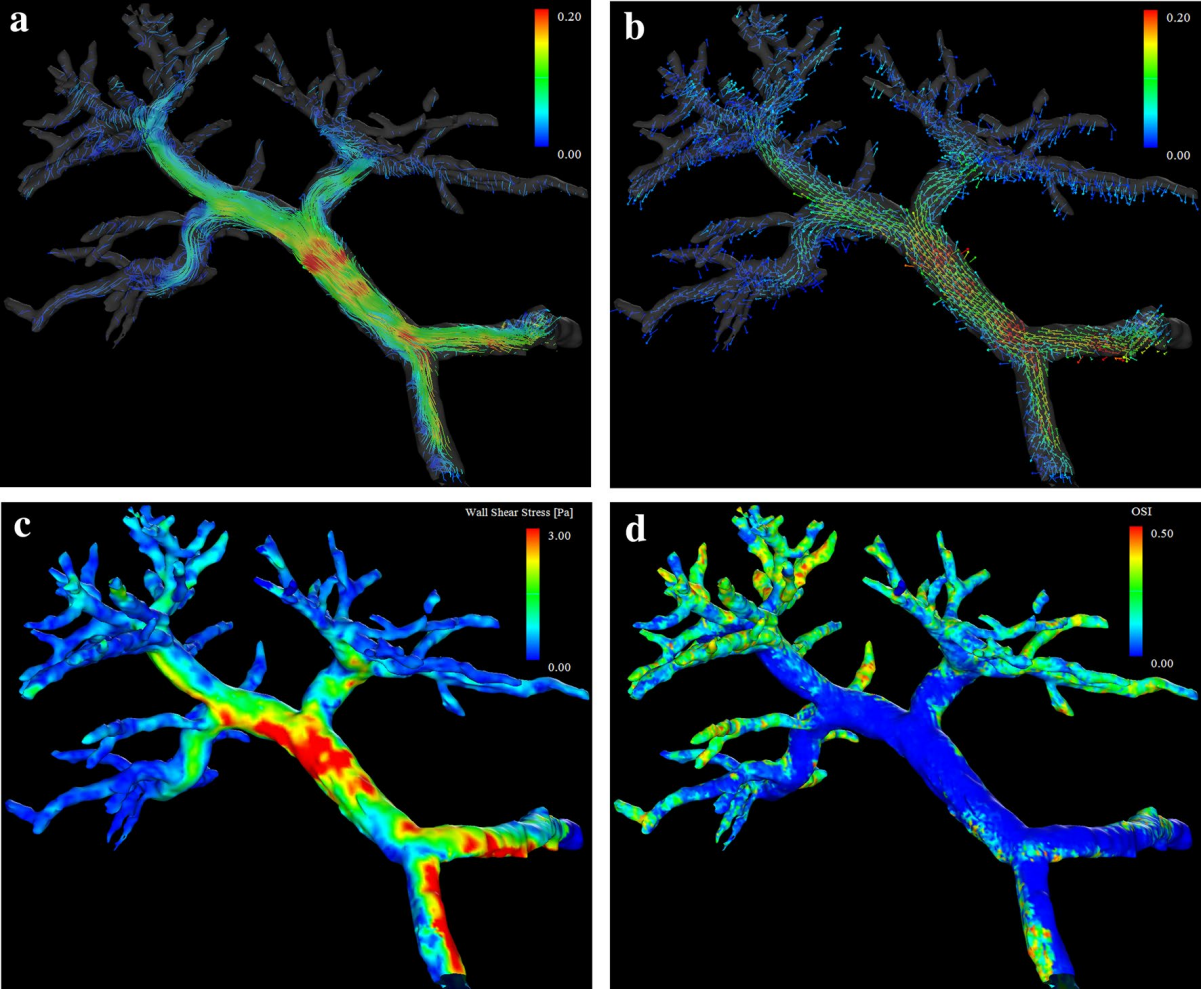
4D Flow MRI acquires the flow velocity vector data all at once, allowing for various types of post-analysis. Same case as Fig. 1. **a** A streamline is lined connecting tandem velocity vectors. **b** A 3D vector field is an assignment of a vector to each point in a subset of space. **c** The wall shear stress (WSS) is defined as the product of the fluid viscosity and the shear rate near the vessel wall. **d** The oscillatory shear index reflects the temporal variation in WSS and is calculated using the temporal variation in the local WSS vector. *OSI*, oscillatory shear index

**Fig. 4:**
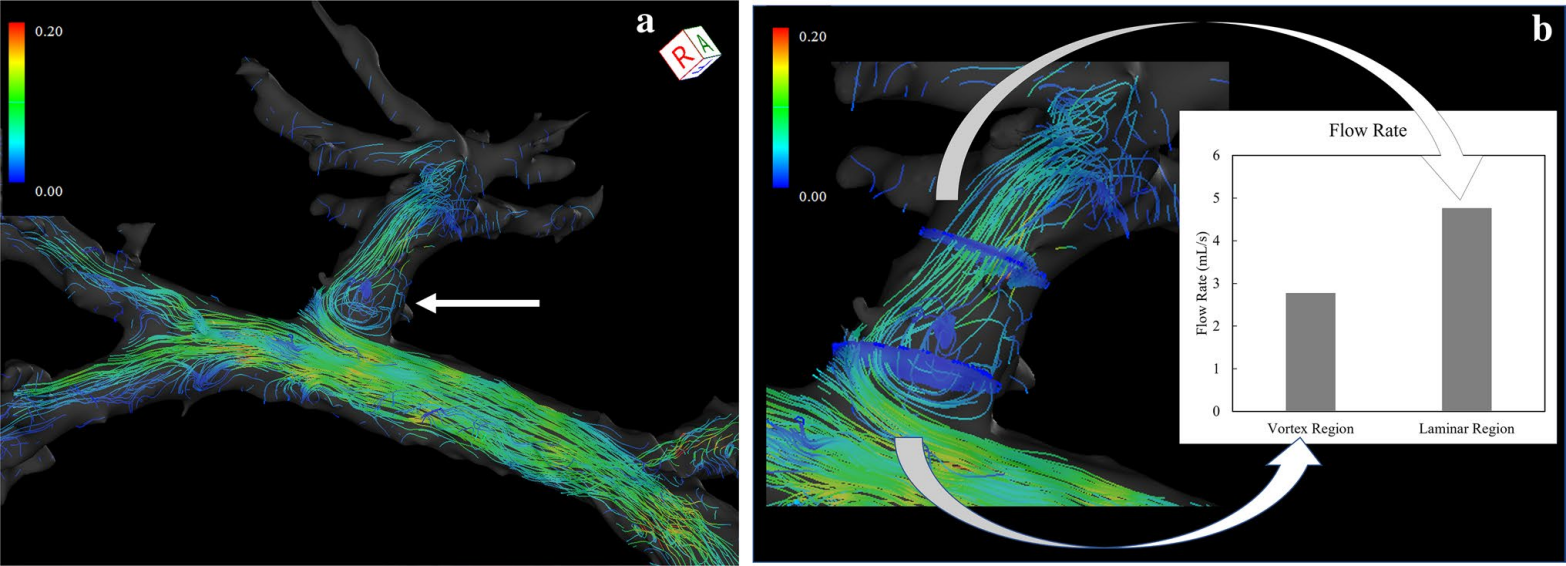
**a** Vortex flow. A vortical streamline is seen at the root of the left portal vein, a finding of vortex flow (arrow). **b** “Retrospective flowmetry” of the vortex flow at the left portal venous root and laminar flow distal to the left portal vein. Blood flow is underestimated in areas of vortex flow than in areas of laminar flow. Doppler ultrasonography and 2D cine phase-contrast MRI cannot retrospectively avoid vortex areas, which is an advantage of 4D Flow MRI

**Fig. 5:**
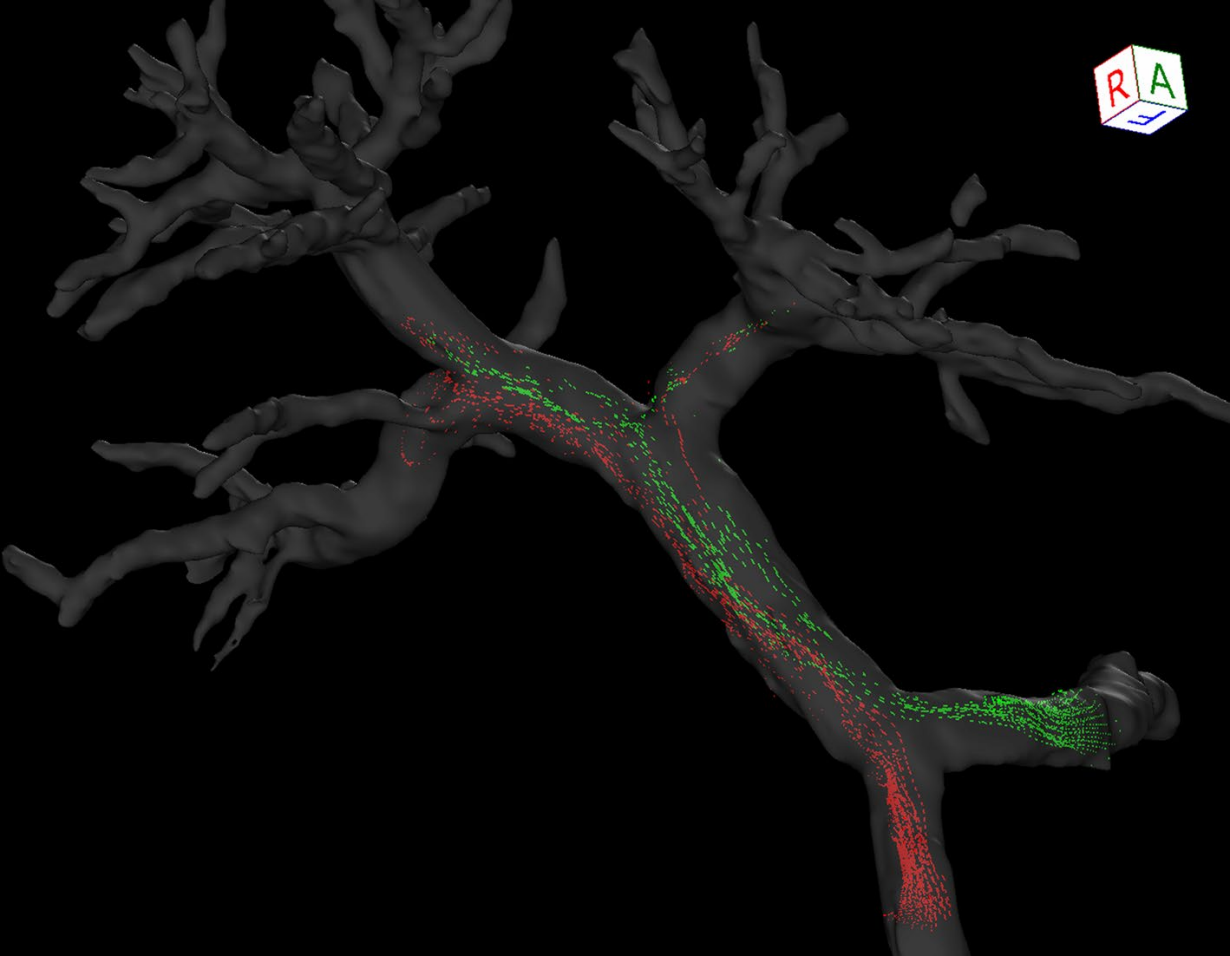
3D pathline image. The red particles flowed from the superior mesenteric vein, and the green particles flowed from the splenic vein. Most of the superior mesenteric venous blood flowed to the right portal vein, and most of the splenic venous blood flowed to the left portal vein and anterior segment branches. This may be useful for the evaluation of the intrahepatic distribution of oral drugs absorbed from the intestinal tract

The post-processed velocity vectors can be displayed on the 3D image, and the overall blood flow can be evaluated using streamlines and 3D vector fields (Fig. 3A and B). This technique is essential for identifying the directions and velocities of the blood flow in the PV area (i.e., hepatopetal or hepatofugal with quantified blood volume). Streamlines are defined as lines connecting tandem velocity vectors [2], which allow visual evaluation of the laminar, helical, and vortical flow and flow velocities by color encoding (Fig. 4A). Helical flow is defined as an antegrade spiral, corkscrew-like motion of blood, whereas vortical flow is defined as a motion of blood that exhibits flow with a direction of rotation that deviates by more than 90 degrees from the physiological flow direction [49]. In addition, selecting arbitrary vessel cross sections and creating a 3D particle trace image, defined as virtual particles tracing the measured three-directional velocities over the cardiac cycle, allows visualization and evaluation of complex blood flows (Fig. 5 and Movie 1) [5, 7, 9].

**Fig. 6:**
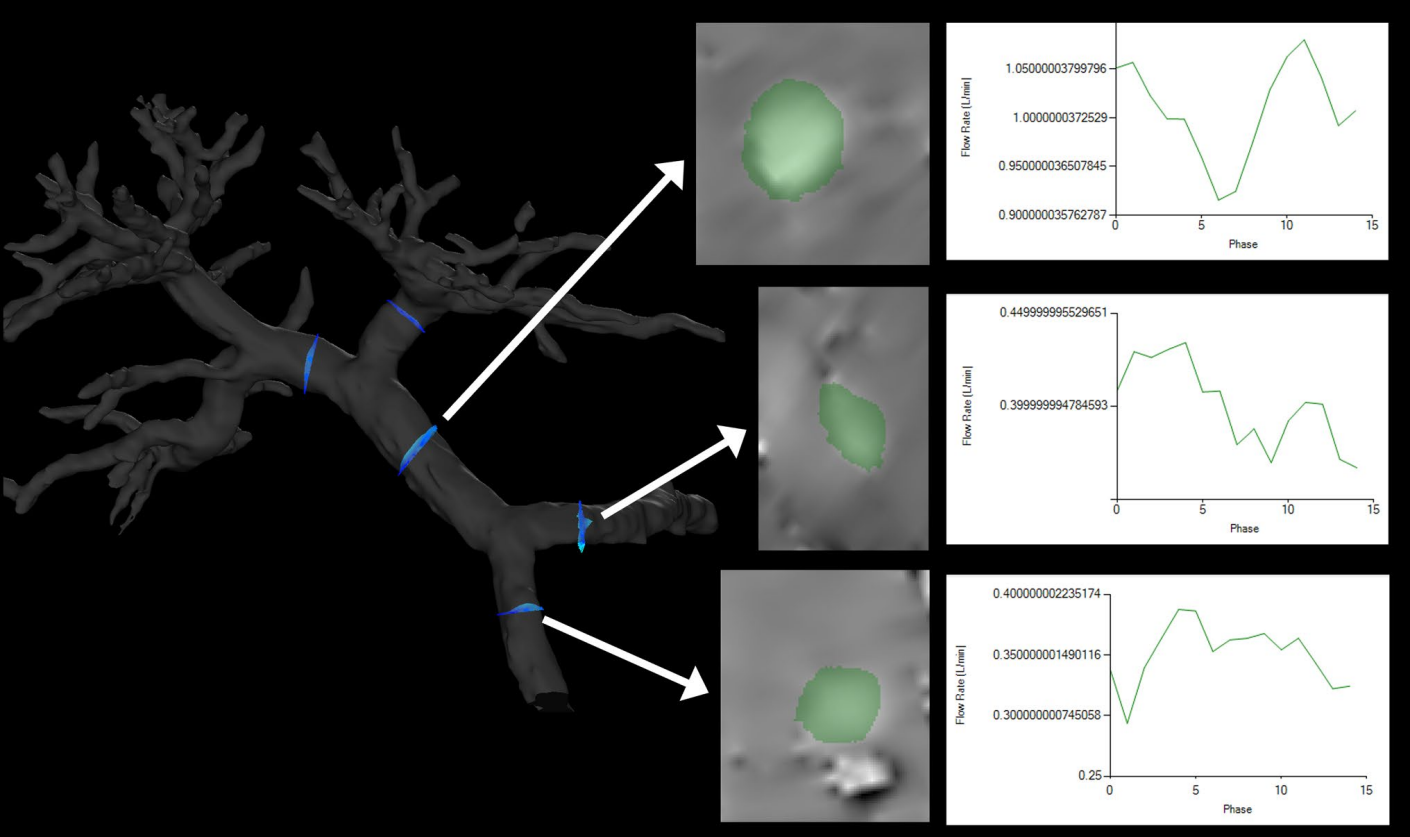
Blood flow analysis. By selecting an arbitrary vessel cross section, the flow velocity vector perpendicular to the flow can be displayed (blue: in-plane vector display). By correcting the misalignment on the phase image (middle), the in-plane flow rate can be displayed for each time phase (right)

Selecting arbitrary vessel cross sections for blood flow analysis allows the display of time-phase-specific flow velocity vectors on those vessel cross sections (Fig. 6). At this time, any misalignment between the vessel geometry and the flow velocity vectors is corrected for each cross section. The area, maximum and average flow velocity, and flow rate of an arbitrary cross section can be measured. In “retrospective flowmetry,” accurate measurement is possible by visually avoiding non-laminar flow areas (Fig. 4B), which is an advantage of 4D Flow MRI over Doppler US and 2D cine PC MRI [50, 51]. Other blood flow parameters that can be evaluated include helicity and vorticity values, which indicate vortex strength, energy loss values, and wall shear stress (WSS) and oscillatory shear index (OSI) values, which provide information on blood flow around the vessel wall (Fig. 3C and D). WSS is defined as the derivative of the fluid viscosity and the shear rate near the vessel wall. OSI reflects the temporal variation in WSS and is calculated using the temporal variation in the local WSS vector. The association of WSS and OSI parameters with vascular lesions has been shown for the aorta [3, 4] and cerebral arteries [52, 53], and these changes have been reported to be associated with atherosclerosis and aneurysm formation. However, no studies have evaluated these aspects in the PV area or abdominal veins, and their usefulness is yet to be fully explored.

### Validation of 4D Flow MRI

Validation of the blood flow data generated by 4D Flow MRI in terms of reproducibility has been reported [54]. In the portal system, assurance compared to US and 2D cine PC MRI has been studied, and high concordance rates have been reported [5, 7, 10, 14, 26]. However, 4D Flow MRI tends to show lower flow velocities than Doppler US, which is attributed to averaging and partial volume effects due to lower temporal and spatial resolution than US, and to the fact that the data are composed of multiple cardiac cycles. Frydrychowics et al. compared portal blood flow on 4D Flow MRI with radial sampling and open perivascular US in pigs and showed that the measurements are in good agreement [39]. We also compared 4D Flow MRI and ultrasound flowmeter measurements in portal vein-assumed steady flow using a straight phantom and found that the measurement error was small [23]. In addition, several studies have evaluated internal consistency as an indirect validation. There are two methods. The first method is to measure multiple locations in the main PV area and evaluate the agreement of the flow rates; Roldan-Alzate et al. evaluated the flow rates at three locations in the main PV area and reported a high agreement rate of 4.2% ± 3.9% for the average absolute error [8]. The second method is to evaluate the degree of flow coincidence before and after the vascular bifurcation, as expressed by the following equation [8, 9, 11, 13]:

$$ {\text{Q}}_{{{\text{SV}}}} + {\text{Q}}_{{{\text{SMV}}}} = {\text{Q}}_{{{\text{PV}}}} = {\text{Q}}_{{{\text{RPV}}}} + {\text{Q}}_{{{\text{LPV}}}} $$

High concordance rates have also been demonstrated in this regard. For example, Roldan-Alzate et al. reported that the error of Q_SV_+Q_SMV_ for Q_PV_ was 5.9 ± 2.5%, showing an excellent correlation (*r*^2^ = 0.99), and the error of Q_RPV_+Q_LPV_ for Q_PV_ was 5.8 ± 3.1%, also showing an excellent correlation (*r*^2^ = 0.99). The interobserver and intraobserver variabilities have also been evaluated, and both have indicated very low bias (interobserver bias, 3%; intraobserver bias, 1%) [8, 11, 43]. The test–retest reproducibility, where the same subject is consecutively imaged to evaluate the reproducibility, has also been studied, and good results have been reported for large vessels [9, 12].

### Limitations and future prospects of 4D Flow MRI

4D Flow MRI has several drawbacks with regard to the portal region. First, although several accelerated imaging methods have been adapted for 4D Flow MRI, the imaging requires a relatively long time in many cases. This is because the imaging requires electrocardiogram gating, respiratory gating, and a large FOV. Furthermore, combining the positioning and morphological imaging, a single session often requires around 1 h [14, 23, 24]. This makes the examination difficult for patients in poor condition or those who have difficulty lying in bed for a long time, and also makes scheduling MRI rooms more difficult. However, the imaging time has been reduced considerably by using accelerated imaging methods, and further acceleration can be expected with advances in equipment and sequences [55]. Second, the spatial resolution remains insufficient. Recent reports on the portal system have shown a near iso-voxel size of around 1.2–1.5 mm [6, 8, 11, 13, 23–25], with a lower spatial resolution than 2D cine PC MRI. A low spatial resolution is reported to cause a partial volume effect, and a low temporal resolution is reported to cause a smoothing effect of the flow waveform, resulting in poor blood flow evaluation [9]. Third, although the contrast media increase the SNR and yield good results when assessing small or tortuous vessels, they cannot be used in the case of renal dysfunction or allergy. Recipients in the early post-transplant period and patients with cirrhosis may have systemic edema, and non-contrast MRI may not provide adequate image quality; therefore, contrast media would be recommended whenever possible, but this is not always possible, as many such patients exhibit hepatorenal syndrome. Fourth, image analysis after imaging requires at least 1–3 h as well as human resources. As reported for the aortic region [48, 56], AI-based segmentation and analysis are expected to be applied to the PV region to reduce the analysis time and make the results uniform by eliminating human variability [55].

## Clinical considerations in 4D Flow MRI for the portal system

### Cirrhosis and portal hypertension: pathophysiology and comparison with healthy subjects

Early investigations of 4D Flow MRI for the portal region focused on the validation of imaging methods using healthy subjects and cirrhotic patients (Table 1) [5–8, 10–13]. Although reports of flow rates in the main PV area using 4D Flow MRI have varied from an average of 10.92 to 18.83 mL/s in healthy subjects [8, 11, 13, 43], they are comparable to those reported previously using 2D cine PC MRI and Doppler US [35, 57–60]. The progressive distortion of the architecture owing to fibrosis and regenerative nodules in the liver with chronic hepatitis and cirrhosis increases the vascular resistance at the sinusoidal level [61–63]. In response, a vasodilator is released to maintain hepatic blood flow, which causes systemic hypotension. This results in a compensatory increase in cardiac output, “a hyperdynamic state,” which in turn increases the portal venous pressure, as the primarily increased splenic venous blood flow further increases the portal venous blood flow [7, 64, 65]. This is accompanied by the development of hepatofugal collateral blood flow [5, 6]. As cirrhosis progresses, the intrahepatic portal branches become narrow, and the blood flow in the PV area is reduced [34]. However, in the case of severe dilatation of the paraumbilical vein, the blood flow in the main PV is increased [5]. 4D Flow MRI can evaluate the morphology, flow direction, and flow rate of these intrahepatic and extrahepatic PV areas and major collateral blood vessels all at once, providing a comprehensive assessment of the hemodynamic changes in each patient [5–7]. In a 4D Flow MRI study comparing healthy subjects, cirrhotic patients showed a hyperdynamic state, with high variability of the portal blood flow among patients, a predominant increase in the vascular area and flow volume of the splenic vein (SV), and increased supraceliac aorta flow velocity [7, 8, 12]. These are consistent with previous studies using other modalities [60, 66]. Brunsing et al. showed that cirrhotic patients with a portosystemic shunt (PSS) had significantly higher supraceliac aorta and PV flow compared to cirrhotic patients without PSS and healthy subjects, which may also indicate the hyperdynamic state in cirrhosis with portal hypertension [43]. 4D Flow MRI can evaluate the arterial and portal regions simultaneously by setting a relatively high VENC (60–250 cm/s), and the hepatic arterial buffer response, which implies a compensatory increase in the arterial blood flow when the PV flow is reduced, can also be confirmed [6, 9, 11, 14, 43]. The response of the PV flow to meals has also been studied previously, and patients with cirrhosis have been reported to have a lower postprandial increase in the PV flow than healthy subjects [67–69]. Roldan-Alzate et al. performed 4D Flow MRI imaging of healthy subjects and patients with portal hypertension before and 20 min after a meal and showed that the postprandial blood flow increase in the portal system was lower overall in patients with portal hypertension than in healthy subjects [11]. They recently reported on the diurnal variation in the portal blood flow by performing multiple time-lapsed imaging [70]. No other method can simultaneously evaluate these physiological or pathological hemodynamic changes in the entire abdomen, which is the main advantage of 4D Flow MRI.

**Table 1:** Imaging methods of 4D Flow MRI in the portal venous system

Subject (No.)	Normal (age)	Stankovic [5]	Frydrychowics [6]	Roldan-Alzate [8]	Landgraf [13]	Roldan-Alzate [11]	Bane [12]	Dyvorne [14]	Stankovic [7]	Stankovic [9]	Brunsing [44]
		18 (28.6±3.1)	0	7 (32.2±10.1)	15 (NA)	6 (32±10)	0	3 (NA)	41 (42.7)	10 (22.3)	21 (50.4)
	Cirrhosis (age)	5 (62.5±13.7)	24 (55.9±10.4)	17 (58.6±6.7)	29 (NA)	12 (54±12)	52 (57)	7 (NA)	20 (57.7)	0	26 (58)
MRI device		3T Siemens	3T GE	3T GE	3T GE	3T GE	1.5T Siemens	1.5T Siemens	3T Siemens	3T Siemens	3T GE
4D-Flow MRI method		Cartesian	Radial/PC-VIPR	Radial/PC-VIPR	Radial/PC-VIPR	Radial/PC-VIPR	Spiral with CS	A. Spiral with CS B. Cartesian	Cartesian	Cartesian	Modified Cartesian
Fasting before examination		NA	NA	3 h	NA	5 h	2–4h	6 h	overnight	6 h	NA
Use of contrast media		none	21 cases	All	All	All	31 cases	7 patients	NA	none	All
Respiratory gate		Navigator echo	NA	Navigator echo	Navigator echo	Navigator echo	Breath hold	A. Breath hold B. Navigator echo	Navigator echo	Navigator echo	Self-navigation
Velocity encoding (cm/s)		50	60	Normal 100 patient 60	60	100 or 120	60	60	50	100	80–250
Acquisition time		16–22 min	10–12 min	10–12 min	10–12 min*	12 min	22 s	A. 22 s B. 11 min 21 s	15–22 min	14.6 min	10–11 min
Time frames per R–R interval		16–19	14	14	14 (time-resolved)	14	NA	12	16–20	12.4	20
Normal PV flow rate (mL/s)		NA	NA	18.33	15.5	18.83	NA	NA	10.92	12.68	16.17
Validation for blood flow analysis		2D cine PC, US	NA	IC	IC	IC	Reproducibility	2D PC, IC	US	IC	IC

PV, portal vein; NA, not applicable; 2D cine PC, 2D cine phase-contrast MRI; US, ultrasonography; PC-VIPR, phase-contrast vastly undersampled isotropic projection reconstruction; IC, internal consistency; CS, compressed sensing; *time-average method took 3–4 min per study

### Cirrhosis and portal hypertension: risk classification for rupture of esophageal varices

**Fig. 7:**
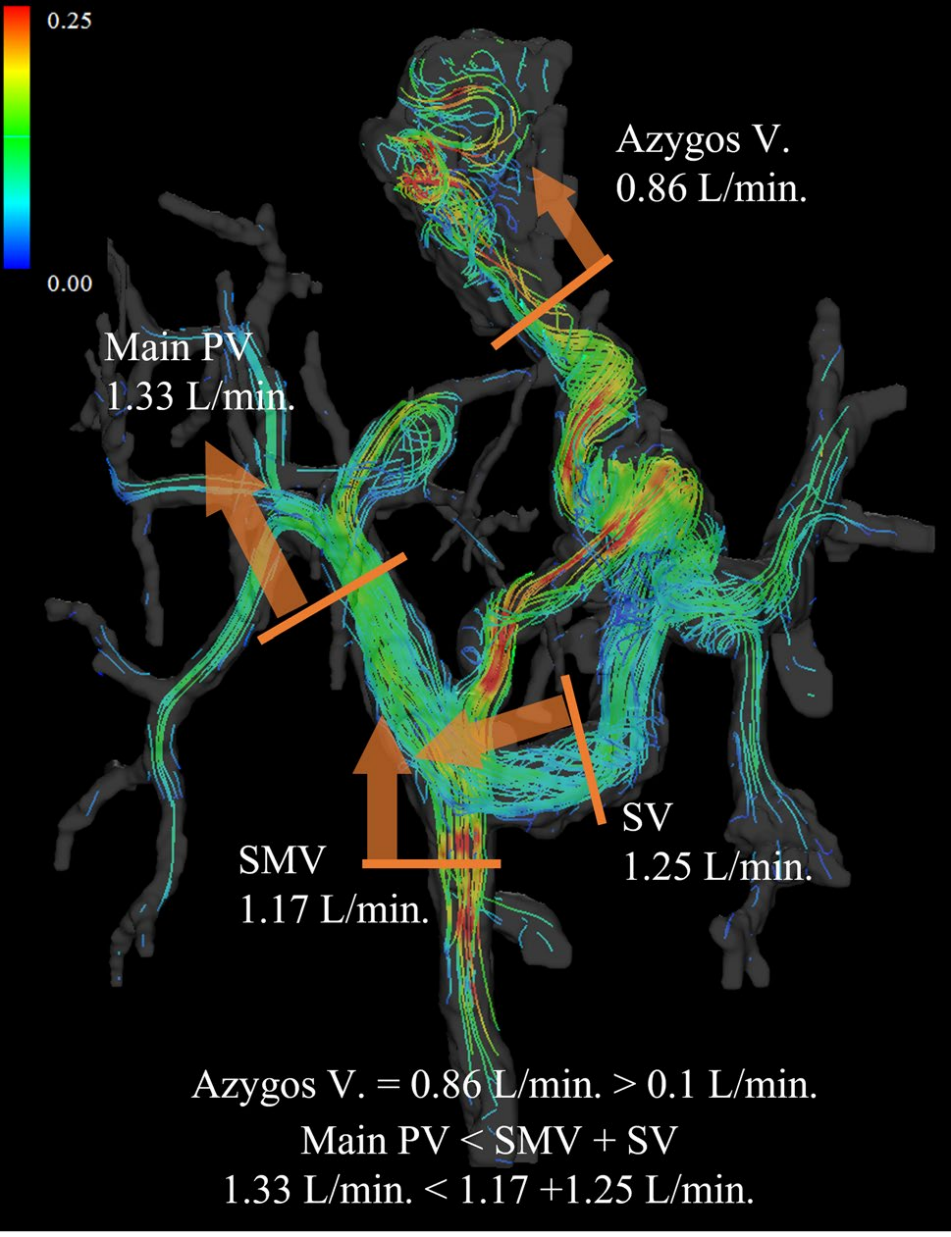
Male in his 30s who were treated endoscopically for ruptured esophageal varices with an underlying primary sclerosing cholangitis. Azygos vein = 0.86 L/min > 0.1 L/min and main portal vein at hilum (1.33 L/min) < superior mesenteric vein (1.17 L/min) + splenic vein (1.25 L/min). Therefore, the patient is considered to be at high risk of rebleeding. *PV*, portal vein; *V*, vein; *SMV*, superior mesenteric vein; *SV*, splenic vein

In patients with cirrhosis, elevated portal venous pressure is an important prognostically relevant symptom that can lead to complications such as varices [71, 72]. Although endoscopy and angiography are adopted as prophylactic and emergency hemostatic methods for esophagogastric varices [71, 73], prior evaluation is important because the prognosis is poor after bleeding. Although several risk assessments for esophagogastric varices rupture have been proposed, endoscopy and the hepatic venous pressure gradient (HVPG) have shown better results [74–77]. Although endoscopy has the advantage of subsequent variceal treatment, few varices require treatment; the procedure causes pain, can lead to complications such as perforation, and is expensive [71, 78]. HVPG is the difference between the balloon catheterized hepatic venous wedge pressure and the free hepatic venous pressure. It is said to indicate an increased risk of variceal rupture at 12 mmHg or higher [13, 71, 75, 77]. Although catheterization provides direct measurement of the pressure, it requires specific expertise, involves some invasiveness, and is expensive, making it unsuitable for routine testing. By contrast, 4D Flow MRI allows non-invasive evaluation and is suitable for repeated evaluations. Although 4D Flow MRI cannot directly evaluate PV pressure, it can evaluate variceal morphology, flow direction, and flow rate, allowing indirect evaluation of portal hypertension [8, 13]. Motosugi et al. compared endoscopic findings of esophageal varices with 4D Flow MRI findings in patients with cirrhosis and reported azygous venous blood flow > 0.1 L/min and fractional flow change in PV < 0 (PV at hilum flow < SMV+SV flow) correlated with high-risk esophageal varices on endoscopy [21]. They reported a sensitivity of 100% and specificity of 100% when both of these findings were present, suggesting that 4D Flow MRI may be useful in assessing the risk of esophageal variceal rupture (Fig. 7). Improvement in portal hypertension with the introduction of nonselective beta blockers has also been reported [61], and visual and quantitative assessment of risk reduction is expected in the future.

### Cirrhosis and portal hypertension: evaluation of blood flow and function of the TIPS pathway

Complications arising from severe portal hypertension include esophagogastric variceal bleeding, refractory ascites, and pancytopenia due to increased splenic function [71, 72]. TIPS is widely used in Europe and the USA to directly reduce portal pressure and mitigate these complications [79–82]. TIPS is a procedure that creates a new shunt between the PV area and the hepatic vein, allowing direct flow from the PV area into the systemic circulation. It can be performed quickly and safely at high-volume centers. After TIPS creation, its shunt function should be evaluated continuously; too many shunts may increase the risk of hepatic encephalopathy (HE) and too few shunts may insufficiently reduce the portal pressure, resulting in the recurrence of portal hypertensive symptoms [20, 79, 82]. Until recently, shunt function has been assessed by clinical evaluation of changes in liver function and portal hypertensive symptoms, as well as by imaging evaluation, such as venography and Doppler US [17]. Venography can measure HVPG and also allows visual evaluation using contrast media. Although it is considered the gold standard, it is invasive, expensive, and not suitable for repeated testing [17]. Doppler US is noninvasive, but it has the aforementioned drawbacks, and comprehensive evaluation is difficult. Another report showed that only 53% of cases with abnormalities noted on Doppler US were diagnosed by venography, indicating that the accuracy is not high [83]. Therefore, 4D Flow MRI has recently been used for visual and quantitative studies [17–20]. Stancovic et al. evaluated abdominal blood flow before and 4 weeks after the TIPS procedure in 11 patients with refractory ascites or variceal bleeding using Cartesian 4D Flow MRI [18]. Bannas et al. also evaluated hepatic blood flow with radial 4D Flow MRI before as well as at 2 and 12 weeks post-TIPS in 7 patients [19]. These results favorably showed the hemodynamics of the TIPS pathway and visually and quantitatively demonstrated an increase in the peak velocity and flow volume of the main PV, SMV, and SV areas after the procedure. The TIPS-to-PV flow ratio, defined as the percentage of portal flow that flows through the TIPS pathway, showed that the TIPS pathway accounted for 80–90% of the portal blood flow in many favorable cases. One study using 4D Doppler US reported that an increase in the shunt fraction correlates with a decrease in the pressure gradient [84]; therefore, the TIPS-to-PV flow ratio measured by 4D Flow MRI may be useful in evaluating the function of the TIPS pathway and predicting complications [19]. Owen et al. studied the utility of 4D Flow MRI for assessing TIPS dysfunction using venography or 6-month clinical follow-up as a reference standard [20]. They reported that in cases that showed both abnormal TIPS pathway velocities (>190 cm/s or <90 cm/s) and focal turbulence in the pathway, pathological stenosis at venography was found with a sensitivity of 100% and specificity of 100%. These reports suggest that 4D Flow MRI is useful for preoperative evaluation and follow-up of the TIPS pathway because it allows evaluation of the TIPS pathway and surrounding portal venous system without US-like blind spots.

### Cirrhosis and portal hypertension: Evaluation of HE and treatment efficacy

**Fig. 8:**
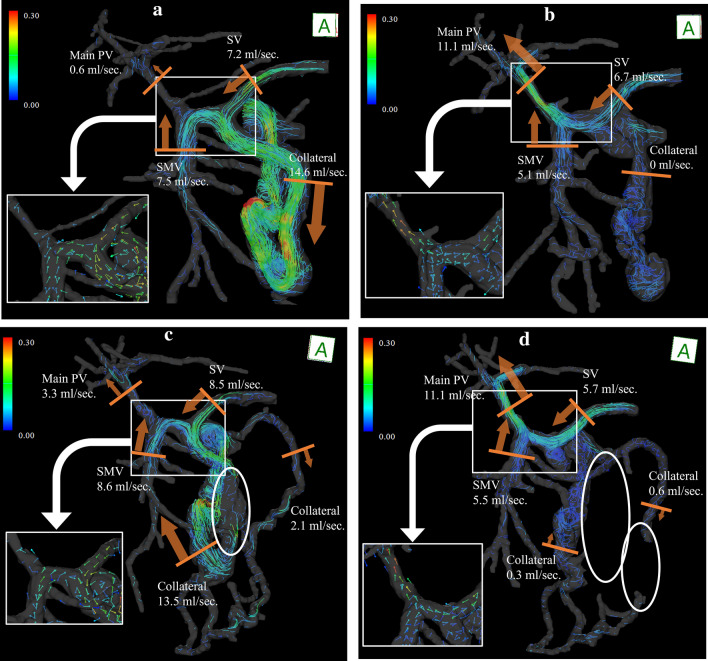
A woman in her 70s presented with cirrhosis and hepatic encephalopathy (HE) associated with large portosystemic shunt (PSS). She also had multiple hepatocellular carcinomas, and transcatheter arterial chemoembolization (TACE) could not be performed owing to portal vein reflux at the previous hospital. **a** Pre-interventional 4D Flow MRI showed a large PSS between the inferior mesenteric vein and the left ovarian vein, which was flowing from most of the superior mesenteric and splenic venous blood flow; the shunt rate of the superior mesenteric vein (SMV) blood flow was 95%, and the serum ammonia level was elevated to 177 μg/dL. This was considered the main cause of HE. **b** Post-interventional 4D Flow MRI. Shunt embolization caused an antegrade change in the superior mesenteric and splenic venous blood flow and increased the blood flow in the main portal vein. The serum ammonia level decreased to 55 μg/dL and HE improved. TACE could be safely performed. **c** Pre-re-interventional 4D Flow MRI. Three months after shunt embolization, the serum ammonia level again increased to 199 μg/dL. The coil embolization site (circle) has lost signal owing to metallic artifacts. However, a large amount of blood flow signal is seen in the PSS again, suggesting reopening. The shunt rate of the SMV was 62%. In addition, a new hepatofugal colonic marginal vein had developed. Portal vein blood flow was reduced, and shunt re-occlusion was performed. **d** Post-re-interventional 4D Flow MRI. Coil embolization area (circle) lost signal. Blood flow in the shunt almost disappeared, and blood flow in the main portal vein increased again. The serum ammonia level improved to 58 μg/dL. Following TACE sessions could be safely performed, and there was no recurrence of HE. *PV*, portal vein; *SMV*, superior mesenteric vein; *SV*, splenic vein

HE is a common liver disease and one of the most debilitating conditions [61, 85]. HE causes brain dysfunction, resulting in impaired consciousness, behavioral abnormalities, and movement disorders. Causes of HE include acute hepatic failure, large PSSs, and liver dysfunction due to cirrhosis. In the case of large PSSs, shunt embolization may improve HE as it prevents undetoxified ammonia and metabolites from flowing directly from the intestinal tract into the systemic circulation [86–89]. We evaluated two cases of HE with large PSS by comparing 4D Flow MRI with angiographic images and clinical findings before and after coil embolization [23]. The streamline of 4D Flow MRI could accurately depict angiographic images and blood flow upstream of the catheter placement, which could not be depicted by angiography. In the cases of HE with large PSS, most of the SMV blood flow was shunted directly into the systemic circulation instead of flowing to the liver, suggesting that it is associated with elevated serum ammonia levels and symptoms of HE. Shunt embolization changed the SMV blood flow anterogradely and hepatopetally and increased the portal venous blood flow. These were clinically consistent with lower serum ammonia levels and improved brain function. Further, we also observed a case in which 4D Flow MRI was useful in the diagnosis of recanalization of large PSS after coil embolization and in the evaluation of re-treatment (Fig. 8; unpublished data). Hence, 4D Flow MRI may be useful not only in evaluating initial embolization but also in evaluating recurrence and re-treatment. Imaging diagnosis of recanalization after coil embolization may be difficult with US or CT owing to deep lesions and metallic artifacts; therefore, 4D Flow MRI may be advantageous.

### Liver transplantation: donor blood flow evaluation

Regardless of the cause of liver disease, liver transplantation is considered to treat end-stage cirrhosis [85, 90, 91]. Living-donor liver transplantation (LDLT) has recently become common owing to the global organ supply shortage [91, 92]. LDLT results in a smaller volume of liver transplanted into the recipient than deceased-donor liver transplantation and a graft-to-recipient body weight of less than 0.8% is associated with an increased risk of the small-for-size syndrome (SFSS) [91–94]. SFSS causes increased portal pressure and perfusion, hyperbilirubinemia, and increased international normalized ratio, leading to graft failure and mortality. Therefore, although transplanting as much liver parenchyma as possible into the recipient is desired, the safety of the donor must be guaranteed, which poses a dilemma. Donors are usually healthy individuals, and surgery is considered safe if more than 30–40% of the liver parenchyma remains, with an average increase in the residual liver size of 80% reported at 3 months postoperatively [93, 95]. However, the degree of postoperative liver enlargement varies among patients and is related to the patient's body size, preoperative liver size, and postoperative residual liver size. In addition, because the remaining vascular vessels are not enlarged postoperatively, the small original vessels increase vascular resistance, exacerbate presinusoidal portal hypertension, and affect the hepatic regenerative process. Therefore, hemodynamic information on the portal system prior to donor surgery is crucial. Rutkowski et al. performed virtual surgery on donor cases using 4D Flow MRI and computational fluid dynamics (CFD) to investigate whether preoperative images could predict postoperative hemodynamics [16]. They reported that by assuming the dilatation of the future residual PV branch obtained from the meal challenge to be the maximum postoperative dilatation, the postoperative blood flow prediction obtained by CFD using preoperative data approximated the actual postoperative streamlines and flow values of 4D Flow MRI. The results indicate that preoperative 4D Flow MRI and CFD may be used to predict postoperative donor hemodynamics. 4D Flow MRI can also simultaneously evaluate the hepatic arteries and veins, which may be useful for appropriate donor selection.

### Liver transplantation: evaluation of recipient hemodynamics, postoperative complications, and treatment efficacy

**Fig. 9:**
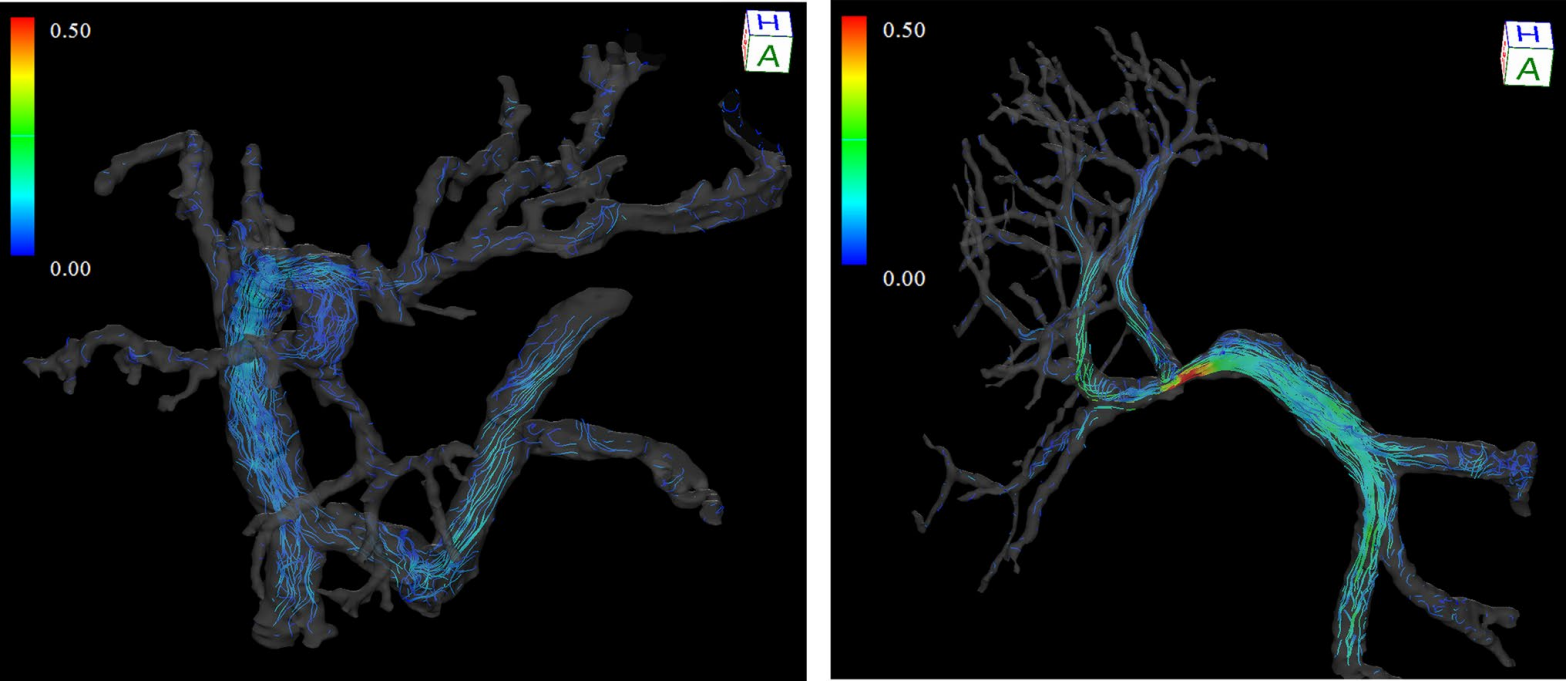
Male transplant recipient in his 50s. **a** The patient had severe atrophy of the right lobe of the liver and compensatory enlargement of the lateral segment of the left lobe owing to cirrhosis of unknown cause. Intrahepatic blood flow was poor except in the medial segment. To and fro blood flow was seen in the portal branches of the lateral segment. **b** 4D Flow MRI one week after transplantation of the recipient's liver right lobe. The right portal vein of the transplanted liver was much smaller in diameter than the recipient's main portal vein. Therefore, jet flow was observed at the anastomosis. The flow velocity in the main portal vein was increased, and a relatively homogeneous distribution of the intrahepatic blood flow was confirmed

MRI of the recipient is challenging. Preoperative massive ascites and subcutaneous edema cause RF wave attenuation and signal loss in the center of the body. Patients are admitted to an intensive care unit after surgery. It is difficult to perform 4D-Flow MRI, which requires a long imaging time, immediately owing to the limitation of their general condition. Meanwhile, accurate evaluation of tortuous portal collateral vessels and deep veins is difficult with US [96], and even if the morphology is known with CT, the blood flow and direction are difficult to assess [22]. 4D Flow MRI is a useful tool for these evaluations (Fig. 9). In the late postoperative period, the patient's general condition is more stable, allowing for safer 4D Flow MRI imaging and evaluation of vascular stenosis and the associated collateral pathway development. Lenz et al. performed noncontrast 4D Flow MRI to evaluate blood flow for esophageal variceal bleeding in a recipient with renoportal anastomosis for PV thrombosis [22]. In this case, Doppler US evaluation was difficult because of the deep location of the anastomotic vessels and collateral vessels; however, 4D Flow MRI enabled comprehensive evaluation of blood flow and contributed to the decision of the treatment strategy. We also evaluated the hemodynamic changes before and after portal stent placement in a patient with PV anastomotic stenosis and presinusoidal portal hypertension after LDLT using 4D Flow MRI [24]. 4D Flow MRI clearly showed turbulent flow distal to the PV stenosis and intrahepatic blood flow disproportion and effectively depicted improved blood flow after stent placement. In addition, the severe hepatofugal collateral flow seen before stenting was markedly reduced in internal streamlines the day after treatment, allowing early determination of the treatment efficacy, which is difficult with morphological images such as CT. No technique other than 4D Flow MRI can comprehensively evaluate the complex hemodynamics after liver transplantation. With this feature, future applications of 4D Flow MRI in evaluating pathological conditions such as SFSS are expected.

### Budd–Chiari syndrome: evaluation of hemodynamics and treatment efficacy

**Fig. 10:**
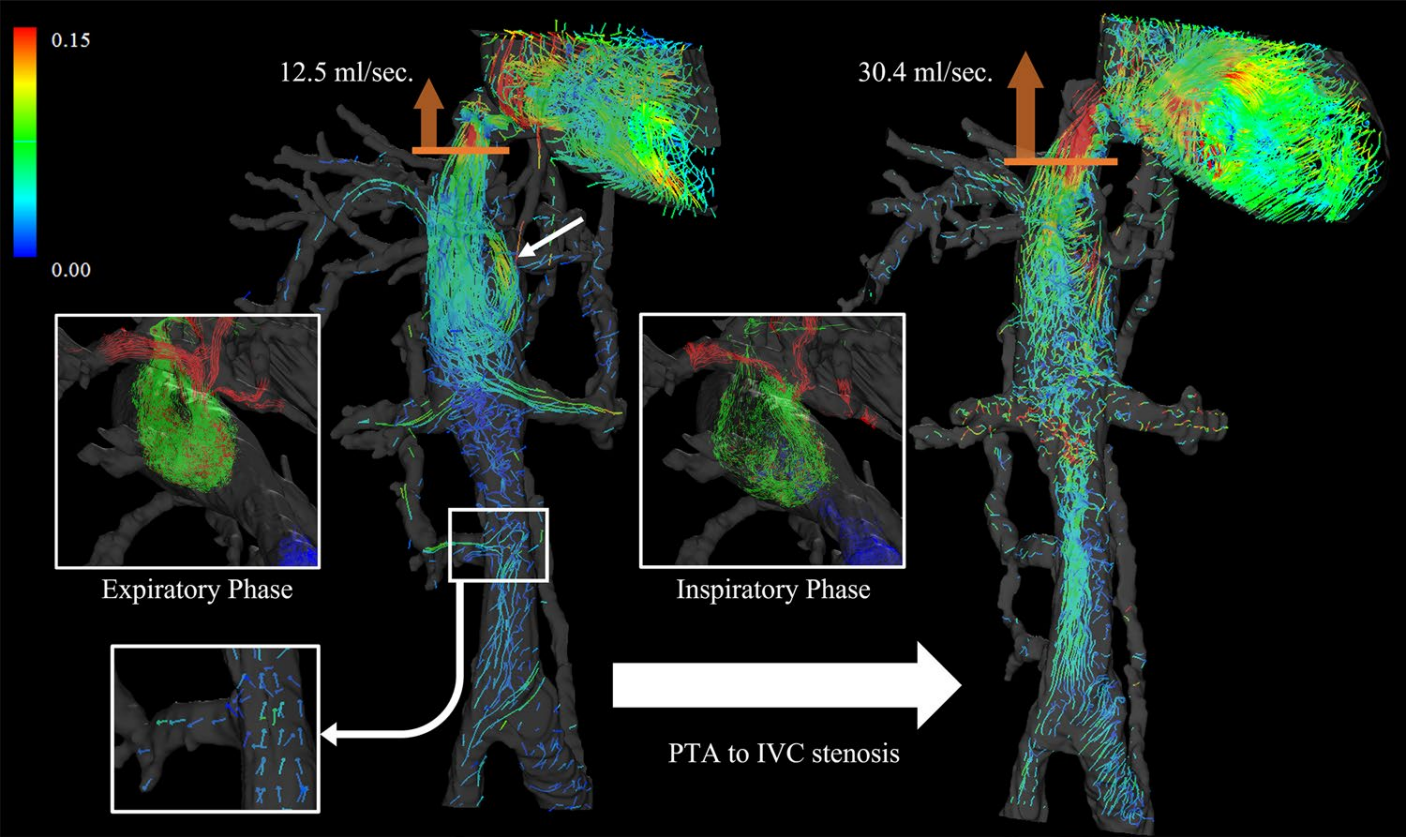
Male in his 50s with Budd–Chiari syndrome. (Left) There is obstruction of the three branches of the hepatic vein, and the hepatic venous blood joins the inferior vena cava (IVC) via a dilated accessory hepatic vein (arrow). Jet flow is seen in the suprahepatic IVC, indicating IVC stenosis rather than obstruction. IVC blood flow below the renal vein is stagnant and mostly flows into the collateral channels (vector view). Two respiratory phases are shown on each side: in the expiratory phase, blood flow from the accessory hepatic vein rotates in the IVC and there is no inflow of infrarenal IVC blood flow; in the inspiratory phase, infrarenal IVC blood flow merges with blood flow from the accessory hepatic vein and flows in an antegrade fashion. (Right) After percutaneous transluminal angioplasty of IVC stenosis. IVC blood flow just below the stenosis increased from 12.5 mL/s to 30.4 mL/s. Stasis of IVC blood flow under the renal vein was also eliminated. *PTA*, percutaneous transluminal angioplasty; *IVC*, inferior vena cava

Budd–Chiari syndrome is a syndrome of hepatic congestion and portal hypertension due to obstruction or stenosis of two or more main hepatic veins or anywhere from the inferior vena cava (IVC) to the right atrium [90, 97]. Chronic Budd–Chiari syndrome develops intrahepatic venous shunts and other collateral veins, such as paravertebral and subcutaneous veins [97, 98]. Hepatofugal collateral vessels associated with portal hypertension, such as esophagogastric varices, are also observed. Various treatments are adopted depending on the causative disease and hemodynamics; interventional radiology includes percutaneous transluminal angioplasty, stenting, and TIPS, for example [90, 97, 99, 100]. We analyzed the hemodynamics of a case of Budd–Chiari syndrome with suspected obstruction of three branches of the hepatic veins and membranous obstruction of the IVC with 4D Flow MRI in two respiratory phases (inspiratory and expiratory phases) (Fig. 10) [25]. 4D Flow MRI made it possible to evaluate the entire abdominal venous and portal system in one series of imaging. The results showed a pore in the membranous portion of the IVC that exhibited a high-flow jet, indicating that this case was not an IVC obstruction but a stenosis. Examination of the 3D pathline image in the inspiratory and expiratory phases showed hemodynamic changes associated with respiration. 4D Flow MRI allowed visual and quantitative evaluation of complex intra- and extrahepatic hemodynamics that were difficult to assess with Doppler US and CT, and contributed to changes in the treatment strategy. Further, the improvement in blood flow after balloon dilation of the IVC stenosis was also quantitatively confirmed by 4D Flow MRI. In the evaluation of venous blood flow, respiration, in addition to the cardiac cycle, has a strong effect [40]. Hence, separating the respiratory phases is expected to provide a more accurate understanding of the disease pathology. However, imaging with separate respiratory phases is still in the development stages and currently requires multiple sessions [25]; therefore, future technological innovations are expected to shorten the imaging time and enable imaging in a single session. In this regard, the self-navigation method may be effective [43]. In addition, because of the high-flow velocity in the jet portion, the dual-VENC method is considered necessary for accurate evaluation in a single session together with the slow-flowing venous stagnation portion [101].

### 4D Flow MRI for children

4D Flow MRI in children involves some difficulties that are not encountered in the case of adults. Because of the relatively long imaging time and physical restraints, sedation or general anesthesia may be required for younger children. In addition, because the vasculature is smaller than in adults, the vasculature that can be accurately evaluated with the current limited spatial resolution is limited to large vessels such as the main PV and SMV areas. However, 4D Flow MRI, which allows comprehensive evaluation without exposure to radiation, may be useful in many situations such as the evaluation of pathological conditions and surgical planning as well as post-treatment evaluation. Several studies have used 4D Flow MRI in the thoracic region for children, including for assessing congenital heart disease [102, 103]. For clinical application in the abdomen, Parekh et al. reported the use of a 1.5 Tesla MRI system [26]. They performed 4D Flow MRI, with general anesthesia if necessary, on 15 normal PV cases and 13 cases of surgical PV shunt creation for PV thrombosis. 4D-Flow MRI for children produced favorable 3D images with a high inter-observer agreement rate (*κ* = 0.67). The image quality was significantly better for children older than 10 years than for children younger than 10 years, possibly because the PV diameter is smaller in younger children [44]. They concluded that 4D Flow MRI of the portal region is feasible in children and is useful for comprehensive 3D image visualization and qualitative assessment.

## Conclusion

4D Flow MRI is a new technology that allows comprehensive, retrospective, and quantitative assessments of the PV flow in normal and pathological cases. With further refinement in related technologies, 4D Flow MRI will be utilized in clinical applications for the abdomen.

## Supplementary Information

Below is the link to the electronic supplementary material.

Supplementary file1 (MP4 13780 KB)

Supplementary file2 (MP4 2141 KB)

Supplementary file3 (MP4 1352 KB)
